# Measured and Perceived Exercise Intensity During the Performance of Single-Task, Cognitive-Motor Dual-Task, and Exergame Training: Transversal Study

**DOI:** 10.2196/36126

**Published:** 2023-02-02

**Authors:** Matthieu Gallou-Guyot, Anaick Perrochon, Romain Marie, Maxence Bourgeois, Stephane Mandigout

**Affiliations:** 1 HAVAE, UR 20217 Université de Limoges Limoges France; 2 3iL Ingénieurs Limoges France

**Keywords:** exergame, dual-task, exercise intensity, heart rate, cognitive load, active video game, physical activity

## Abstract

**Background:**

The physical and cognitive loads borne during exergaming may differ from more conventional cognitive-motor dual-task trainings.

**Objective:**

The aim of this pilot transversal study was to compare objectively measured and perceived exercise intensity during exergame, cognitive-motor dual-task, and single-task training sessions.

**Methods:**

We recruited apparently healthy young adults who carried out one session of each type of training: exergaming, cognitive-motor dual-tasking, and single-tasking. We used a custom-made exergame as support. The sessions lasted 30 minutes, were spaced at least 24 hours apart, and took place in random order for each group of 4 participants. We used heart rates to assess exercise intensity and the modified Borg scale to assess perception of intensity. In all, 16 apparently healthy young participants carried out all sessions.

**Results:**

There was no difference between the different types of training in mean heart rates (*P*=.27), peak heart rates (*P*=.50), or Borg scale scores (*P*=.40). Our custom-made exergame’s objectively measured and perceived physical load did not differ between cognitive-motor dual-task and single-task training.

**Conclusions:**

As a result, our exergame can be considered to be as challenging as more traditional physical training. Future studies should be conducted in older adults with or without cognitive impairments and incorporate an assessment of cognitive performance.

## Introduction

Exergames (EGs), or “active video games,” are video games played on a digital device, including a wide range of interfaces [1] that require physical activity when played [2]. EGs are a singular form of cognitive-motor dual-task (CMDT) training, which is known to be efficient in terms of both cognitive and physical functions [3,4]. In addition, EGs are considered fun and are enjoyed by most users, as they facilitate exercise in an attractive, motivating, and interactive environment [5]. However, EGs must be sufficiently demanding to induce effects, with regard to cognitive as well as physical loads [2,6], and their efficiency depends to some extent on their intensity.

As a singular form of CMDT training, EGs should not differ in terms of the physical level of solicitation. The tasks players are asked to perform are similar, one of them being cognitive and the other physical, with the difference residing in the support used. Previous studies have shown that most EGs induce moderate [7-10] to vigorous [8,9,11] exercise intensity in healthy young adults. However, there seem to be large variations in intensity between different EGs, depending on the hardware (primarily Kinect or Wii) or game used [11,12]. For example, EGs such as Wii Bowling induce low-intensity physical activity [8], whereas Kinect Boxing or “Your Shape Fitness Evolved 2012” indeed induce vigorous physical activity [9,11]. Variability in exercise intensity between tailor-made EGs is likely to be even greater.

Significant heterogeneity in exercise intensity during exergaming raises questions about the impact of the support used. Would exercise intensity be the same during similar physical training sessions presented in either single-task (ST) or CMDT conditions, whether using a tailor-made EG as a support or not? To our knowledge, no study has compared exercise intensity during exergaming and ST training, and only one study has compared exercise intensity during CMDT and ST training in healthy young adults [13], showing higher intensity under CMDT than ST conditions.

To date, we have been unable to conclusively determine the impact of the training support (ie, direct comparison between exergaming and CMDT training) or the impact of a concurrent cognitive task (ie, direct comparison to ST conditions, considered as the reference modality for physical training) on the level of physical solicitation in healthy young adults. The aim of this pilot transversal study was to compare objectively measured and perceived exercise intensity during EG, CMDT, and ST training sessions.

## Methods

### Participants

Apparently healthy students from the University of Limoges volunteered for this pilot transversal study. The inclusion criteria were young adults aged between 18 and 35 years and fluent in French. The exclusion criteria were having a contraindication to physical activity or having eaten or drunk during the previous 2 hours.

### Ethical Considerations

In accordance with the Declaration of Helsinki as revised in 2013, the volunteers had received an information document detailing the protocol and gave their written consent to participate.

The study was carried out as part of student work in initiation to research during the year 2021. According to French regulations (Jardé law) [14], ethics review was not required.

### Procedure

**Figure 1:**
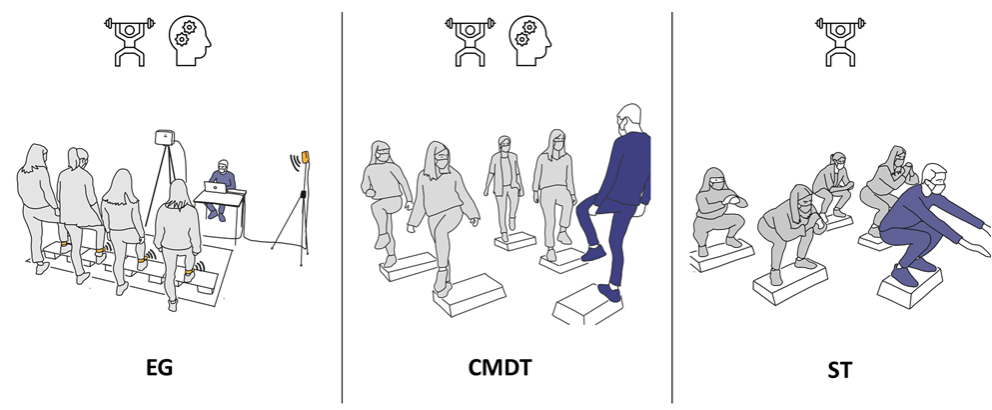
Illustration of the differences between exergame (EG), cognitive-motor dual-task (CMDT), and single-task (ST) training, during the performance of a stepping task. The instructor is represented in blue.

Participants were recruited during practical exercise courses. They carried out 3 types of training: EG, CMDT, and motor ST. The training sessions were designed identically: physical exercises (ST), with concurrent cognitive tasks (CMDT), using an EG as support (EG). The exercise sessions were organized in groups of 4 participants, and the order of training was randomized for each group. They lasted 30 minutes and were spaced at least 24 hours apart. They were divided into eight 3-minute-long exercise sequences (see Table 1), with 30 seconds of rest and instructions in between; therefore, effective physical activity lasted approximately 24 minutes. An instructor supervised, giving instructions and assisting the participants. Sessions were suspended in the event of an injury, significant pain, or heart rates above 90% of the theoretical maximums [15]. We have illustrated the differences between EG, CMDT, and ST training during stepping performance in Figure 1.

**Figure 2:**
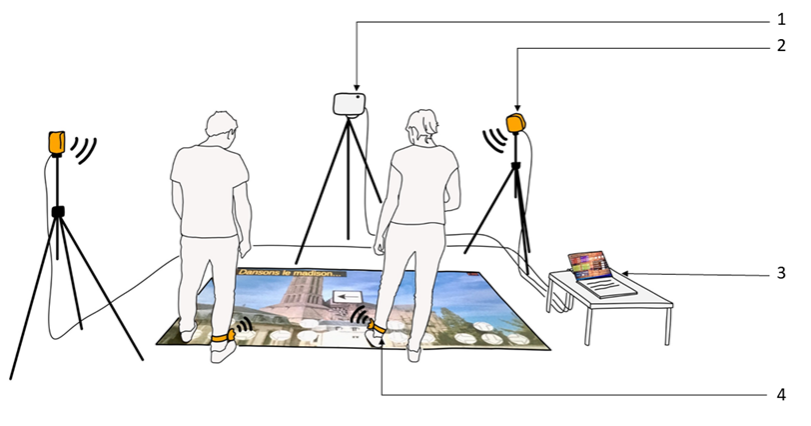
Illustration of the exergame. (1) Video projector, (2) HTC Vive infrared cameras, (3) computer, and (4) HTC Vive trackers. In this example, only 2 participants are performing a stepping exercise.

During EG training, sessions consisted of multiple dual tasks using a custom-designed EG as support (Figure 2). This EG was developed in the *Handicap, Activités Vieillissement, Autonomie, Environnement* laboratory, using the “Virtual Carpet,” which is associated a video projector and HTC Vive cameras and trackers, as the play area [16-18]. The projected scene was a schematic city, and players were asked to move to different points of interest during exercises. Detection by the Vive trackers of a player’s position at a point of interest triggered a change of scene and launched a mini-game, which was carried out by all the players. The motor tasks were mainly stepper, muscle strength, and balance activities. The cognitive tasks were verbal fluency, arithmetic, mental inhibition and flexibility, visuospatial memory, processing speed, and planning. Some exercises required gymnastic equipment (stepper, Swiss or medicine balls, chairs). The training sessions were designed to match with the American College of Sports Medicine [19] and the World Health Organization [20] guidelines and recommendations on physical activity, as well as the recommendations designed to prevent falls in older adults [21]. Details on the exercises are indicated in Table 1.

During the CMDT training, the participants used the same gymnastic equipment, and the sessions were made up of the same associations of cognitive and motor dual tasks as in the EG group but without using the EG (see exercise details in Table 1).

Finally, ST training required the same gymnastic equipment and the same motor tasks as those carried out during EG and CMDT training but without a concurrent cognitive task (see exercise details in Table 1).

**Table 1:** Details of the exercises proposed during the different types of training.

Training	Stepper (with and without a step)	Visuospatial memory and balance	Muscular strength and coordination
EG^a^	Arrows are displayed successively on the projected scene, and participants must reproduce them on a pad with 1 foot, 2 feet, doing a squat, a lunge, etc. The additional cognitive tasks are to not reproduce an arrow (go or no go), to invert them (mental flexibility), or to render them with a time lapse (working memory).	Eight elements displayed within the projected area will turn on and off at a fixed frequency, constituting a growing span. Participants must memorize this sequence and then recall it while moving around. At the same time, they perform motor exercises (knee raising, buttocks to heels, squats, lunges, and jumping jacks). The additional cognitive tasks are to not consider one of the icons or to recall the span from the end.	Participants must perform muscle-strengthening exercises (eg, squat and lunges). At the same time, they must (1) solve mental arithmetic exercises appearing on the projected scene; (2) alternate the exercises performed according to the images appearing on the projected scene; and (3) perform a “categories” game or build a word giving a letter, one at a time.
CMDT^b^	The instructor shows a sequence of movements that the participants must reproduce in mirror (step, squat, lunge, etc). The additional cognitive tasks are to not reproduce an arrow (go or no go), to invert them (mental flexibility), or to render them with a time lapse (working memory).	Participants must displace a total of 8 plots, one at a time. The plot-placing order constitutes the span. Participants must memorize this sequence and then recall it while moving around. At the same time, they perform motor exercises (knee raising, buttocks to heels, squats, lunges, and jumping jacks). The additional cognitive tasks are to not consider one of the icons or to recall the span from the end.	Participants must perform muscle-strengthening exercises (ie, squats and lunges). At the same time, they must (1) alternate the exercises performed according to the auditory or visual stimuli given by the instructor and (2) perform a “categories” game or build a word giving a letter, one at a time.
ST^c^	The instructor shows a sequence of movements that the participants must reproduce in mirror (step, squat, lunge, etc). There is no additional cognitive task.	The instructor demonstrates static and dynamic balance exercises to perform. The participants must perform movements of the limbs and trunk and pass a medicine ball while standing on a Swiss ball or standing on one leg. There is no additional cognitive task.	Participants perform a game mixing together muscle strengthening of the lower limbs and motor coordination by dribbling with a ball. There is no additional cognitive task.

^a^EG: exergame.

^b^CMDT: cognitive-motor dual-task.

^c^ST: single-task.

### Outcomes

The primary outcome was the objectively measured exercise-intensity level assessed in terms of mean and peak heart rates (HRs) during training. We used HR because it is considered the most practical parameter to monitor, especially as regards reliability, safety, and cost [22]. We used validated chest HR Polar H10 monitors (Polar Electro Oy) [23], measuring the participants’ HR_mean_ and HR_peak_ during the 3 types of training.

The secondary outcome was the rating of perceived exertion, using a validated modified Borg scale [24] completed by participants at the end of each training session.

### Statistical Analysis

Quantitative variables were described according to mean and SD or median and IQR. The normality of data distribution was assessed using Shapiro-Wilk tests. Comparison between HR_mean_, HR_peak_, and Borg scale scores during the different trainings (EG, CMDT, and ST) was carried out using a 1-factor ANOVA or a Friedman test according to the normality of the variables concerned. Statistical analysis was performed using RStudio (Rstudio, Inc), and result significance was set at *P*<.05.

## Results

In all, 16 apparently healthy young participants volunteered for the study (6 males, 10 females). Sociodemographic characteristics are presented in Table 2.

Although mean and peak HR variable distribution was normal during the 3 types of training, the Borg scale scores were not. There was no difference between the different types of training (EG, CMDT, and ST) for any of the variables studied: HR_mean_ (*F*_2,45_=1.33; *P*=.27), HR_peak_ (*F*_2,45_=0.7; *P*=.50), and Borg scale scores (*χ*^2^_2_=1.85, *P*=.40; see details in Table 3).

**Table 2:** Participant characteristics.

Characteristic	Value, mean (SD)
Age (years)	24.6 (3.1)
Height (m)	1.72 (0.11)
Weight (kg)	69.1 (15.1)
BMI	23.1 (3.2)
HR^a^_resting_ (bpm^b^)	66.9 (10.4)
HR_max_ (bpm)	187.2 (1.2)

^a^HR: heart rate.

^b^bpm: beats per minute.

**Table 3:** Results depending on type of training.

Result	EG^a^	CMDT^b^	ST^c^
Borg scale score (out of 10), mean (SD)	3.1 (1.4)	3.6 (1.3)	3.3 (1.0)
HR^d^_mean_ (bpm^e^), mean (SD)	119.8 (12.2)	123.8 (16.1)	128.1 (14.7)
Percentage of theoretical HR_max_ (%)	64	66	68
HR_peak_ (bpm), mean (SD)	157.9 (10.1)	163.0 (12.8)	161.7 (14.5)

^a^EG: exergame.

^b^CMDT: cognitive-motor dual-task.

^c^ST: single-task.

^d^HR: heart rate.

^e^bpm: beats per minute

## Discussion

This pilot transversal study was aimed at comparing objectively measured and perceived exercise intensity during EG, CMDT, and ST training sessions in healthy young adults. We observed no differences in mean or peak HR or perceived exertion between the 3 types of training.

### Perceived and Measured Physical Activity Intensity

In our study, physical activity intensities during EG, CMDT, and ST training sessions did not statistically differ. This finding is discrepant with the results of a previous study, which showed higher HR_mean_ under CMDT than ST training [13]. Our results even seemed opposed, with a tendency toward higher HR_mean_ during ST training of nearly 10 beats per minute, close to 70% of theoretical HR_max_, and moderate intensity [25]. This difference with our study may be due to the nature of the requested tasks. Exercise intensity varies considerably according to type of movement, the body part involved (upper or lower limb), the level of difficulty of a given game (frequency and speed), or the participants’ previous experience as players [26-28]. In the study by Herold et al [13], subjects were asked to carry out squats under both conditions while additionally counting backward in CMDT training[13]. In our study, the physical and cognitive tasks were not only more varied but also and above all more complex (see Table 1). In addition, it is important to note the impact that different interfaces can have when comparing interventions. For instance, the quality of immersion and interaction highly impacts the user experience and the usability of immersive EGs [29]. We tried to reduce these effects of complexity and interface by proposing similar and transferable exercises between the different types of training. As a result, the exercises carried out in this study during training sessions were the same in EG, CMDT and ST conditions, with the differences consisting solely of the concurrent cognitive task and the use of a given game as support. In fact, instruction comprehension and error correction were time-consuming, decreasing effective exercise duration and HR over the course of the 30-minute sessions. Understanding more complex combined tasks during CMDT training along with adaptation to new game rules and to a new environment during EG may have been more time-consuming than during ST training.

Previous studies assessing the level of solicitation during exergaming in healthy young adults through HR have shown moderate [7-10] or vigorous [8,9,11] exercise intensity, with highly pronounced variability between studies according to the software or hardware used. Our custom-made EG seemed to induce moderate exercise intensity, equivalent to the physical exercises requested under CMDT or ST conditions. This is a relevant and interesting finding; since exercises under ST conditions are considered as the reference modality for physical training, their equivalence during exergaming and dual-task training enabled us to consider our EG as sufficiently soliciting to induce physical results, while adding a cognitive load in a playful environment.

Moreover, perceived physical intensity assessed through the Borg scale did not differ between the 3 types of training and was globally moderate. The perception of effort by participants in this study was correlated with the results obtained from the percentage of theoretical HR_max_ [25]. This is in line with previous studies showing that adults correctly estimate their less vigorous physical efforts [25,30].

### Limitations

The first limitation of this study is the sample, for which no a priori sample size was carried out. However, our population was homogeneous and showed relative stability in physical intensity. This pilot study constitutes a proof of concept specific to our EG in a highly specific context and population. Consequently, the small extension of the knowledge provided by our study is specific, which complicates its extrapolation to other forms of EGs or populations.

A second limitation is due to the assessment exercise intensity modalities. In this study, given its reliability, safety, and cost, we used HR as a reflection of exercise intensity, whereas other studies have used more direct parameters such as oxygen consumption [8,31] or blood lactate responses [32] to assess exercise intensity during exergaming. Moreover, the software we used did not enable us to analyze HR in detail. It would be relevant to estimate the HR profile to determine whether it remains stable and close to the mean or varies with peaks and rest. Lastly, the assessment of effective HR_max_ rather than theoretical HR_max_ would have made it possible to evaluate reserve HR, thereby limiting impact of interpersonal variability.

### Future Studies

Most of the EGs in which the intensity was assessed in previous studies were commercial video games [7-11]. This is broadly the case, especially insofar as commercial active video games are frequently used in rehabilitation [33]. The main consequence is a lack of control over qualitative and quantitative physical and cognitive exercises. Tailor-made software using mainstream hardware is designed to suit the specific needs of a given audience [34] while applying an easily available and relatively inexpensive solution. For example, the training of older adults presents specificities, in both form and in substance. Understanding is maximized through slow animations, large fronts, and simple rules [35]. With age, physical and cognitive capacities decline while cognitive-motor interference increases [36,37]. Fall prevention in older adults should focus on strength, postural control, stepping, and gait training [38-40], along with mental inhibition and flexibility, processing speed, and visuospatial memory [41-43]. In fact, a fall prevention training protocol with our EG seems possible and relevant, insofar as it facilitates the simultaneous training of cognitive and physical functions. By applying our pilot study’s values and methodology, future studies may assess the extent to which and the efficacy with which our EG solicits in older adults. In addition to its pronounced beneficial effects, our EG may help promote physical activity [44], which is a point of major importance among older adults.

In this study, we investigated physical aspects, not cognition. However, cognitive efficiency and HR may be correlated; a meta-analysis found that participants with better cognitive task results often demonstrated greater cardiac parasympathetic control than those with poorer cognitive performance [45]. This question could be investigated using hybrid systems, coupling functional near infrared spectroscopy, and electroencephalogram headsets [46].

### Conclusion

This pilot transversal study showed that a custom-made EG that could induce moderate perceived and objectively measured exercise intensity, equivalent to CMDT and ST training in healthy young adults. As a result, our EG can be considered as being as relevant as more traditional physical training with regard to exercise intensity. Future studies should investigate the cognitive and physical level of solicitation of our EG in older adults, who are likely to draw benefit from CMDT training.

## Abbreviations

CMDT: cognitive-motor dual-task
EG: exergame
HR: heart rate
ST: single-task
